# Depressive symptoms faced by non-native international medical students in China during COVID-19

**DOI:** 10.3389/fpsyg.2022.1037786

**Published:** 2022-11-17

**Authors:** Gao Xiang, Muhammad Ishfaq Ahmad, Weiqing Zhuang, Ramiz Ur Rehman, Muhammad Akram Naseem

**Affiliations:** ^1^Department of Public Utility Management, Guangzhou Medical University, Guangzhou, China; ^2^Lahore Business School, The University of Lahore, Lahore, Pakistan; ^3^School of Economics and Trade, Fujian University of Technology, Fuzhou, China; ^4^School of Business, Sohar University, Sohar, Oman

**Keywords:** depressive symptoms, pandemic, language barrier, students, financial issues

## Abstract

This study examines depressive symptoms faced by non-native international medical students studying in China during the COVID-19 pandemic. The targeted population for this cross-sectional study included non-native medical students studying in Chinese universities. This study used convenience sampling. An online, self-administered questionnaire was distributed to international medical students studying in Chinese universities from February 2020 to June 2021. The questionnaire collected demographic data, information regarding struggles faced, and used the CES-D-10 Likert scale to assess both the challenges and depression symptoms, respectively. By analyzing the 1,207 students’ responses, the study found that students with poor Chinese language were two times more likely to suffer from depressive symptoms (OR = 2.67; value of p 0.00). Moreover, female students were found more prone (76.35%) than their male counterparts (44.96%). The study found that food adaptability, health issues, accommodation issues, and financial issues were related factors contributing to depressive symptoms among non-native international medical students during the COVID-19 pandemic. The study tried to highlight the factors that resulted in depressive symptoms among non-native international medical students, and the findings may help diplomatic representatives take necessary actions to help their citizens during this difficult time.

## Introduction

The internationalization of higher education has become popular and important due to the influence of globalization since the beginning of the 20-first century. Recently, there has been a rapid increase in students studying abroad worldwide. The Chinese government has promoted world connectivity through educational reforms and welcomed international students to China (Latief and Lefen, 2018). The Chinese government has attracted international students by offering scholarships such as the Chinese Government Scholarship, Chinese Academy of Science, Confucius Scholarship, and Provincial Government Scholarship (Chinese Ministry of Education) (China Scholarship Council, 2009, China)^1^. Additionally, many professors provide financial assistance to international students through research funding.

According to a report published by the Organization for Economic Co-operation and Development (Platise, 2019), international students increased from 3.3 million in 2007 to 5.3 million in 2018. China is among the top 10 host countries for international students (Higher Education Student Affairs 2018–2019). The number of international students being hosted in China is constantly growing (Chow, 2011).

Adjusting to a new educational and social environment can be a challenging and stressful process. Most international students face more severe stress due to culture-shock (Church, 1982) along with other sociocultural factors that are directly linked to the adjustment process (Luzio-Lockett, 1998). The disease caused by the coronavirus (COVID-19), starting in 2019, makes this adjustment much more complex and stressful for international students (Tozini and Castiello-Gutiérrez, 2022). Apart from the typical stressors of attending an educational institution, international students encounter events and stressors beyond those of native students, thereby impacting their mental health (Jamilah et al., 2020). They manage these difficulties without family and familial support. Most importantly, language barriers may result in anxiety or depressive symptoms among students (Parray et al., 2020).

Upon entering their host country, international students begin the process of acculturation through multicultural group interactions (Berry, 2005). This process may require alterations in an individual’s beliefs, identity, or behavior (Abraído-Lanza et al., 2004; Thomson and Hoffman-Goetz, 2009). Similarly, limited language proficiency may result in acculturative stress (Fritz et al., 2008; Shadowen and Terplan, 2019). Because limited language proficiency affects international students’ effective communication, they may not seek help (Prieto-Welch, 2016), resulting in further mental health problems (Kirmayer et al., 2011; Brisset et al., 2014).

Mental health is “a state of well-being in which the individual realizes his or her abilities, can cope with the normal stresses of life, can work productively and fruitfully, and can make a contribution to his or her community” (Wu et al., 2015). Depression during a pandemic is common among students (Hasanah et al., 2020; Rahmasari et al., 2021). It is widely accepted that stressful events influence the individual’s psychological and physical well-being (Dohrenwend and Dohrenwend, 1974; DeLongis et al., 1988). The COVID-19 pandemic is among the most stressful events in history. The fear and uncertainty due to COVID-19, following the lockdowns, strict policies, and social distancing, has accelerated depression and health issues throughout societies (Jamilah et al., 2020). Different factors contributed in this regard as COVID-19-related news adversely affects people’s mental health (James et al., 2020). Similarly, students’ COVID-19 illness perception, including anxiety and depressive symptoms, affected their mental health (Baron et al., 2017). The COVID-19 pandemic has compounded these challenges for the international student community.

Hawkins (2021) highlighted the challenges faced by international students during COVID-19. She found that most international non-native students live in isolation in dorms or apartments, they do not know about the possibility of ordering groceries online, and most students do not have private transportation. As China is no exception, similar issues were faced by international students in China during COVID-19. Even under normal circumstances, international students are more prone to depressive symptoms (Khawaja and Dempsey, 2007; Nguyen et al., 2019; Li et al., 2021). Previously, the relationship between COVID-19 and depression has been studied in the context of medical staff (Chen et al., 2020; Liu et al., 2021) and among the general population, (Salari et al., 2020) but the existing literature is scarce in lieu of the role of the language barrier and other factors in students’ depressive symptoms. To fill this gap, this study aims to identify the effects of sociocultural environment factors, financial security, and curriculum structure on the depression of non-native international medical students in China during the COVID-19 pandemic. This study is essential to the interests and well-being of not only non-native international medical students but also for the global image of China’s higher education.

The paper is organized as follows. The following section traces the Materials and methods of the study, and also provides a description of the data and variables used. Section 3 discusses the results of the study. Section 4 concludes the study and highlights the future research directions.

## Materials and methods

This study targeted non-native international medical students studying in China. The data was collected from February 2020 to June 2021 from different medical universities across China. The questionnaire was in English as the study targeted. The study followed a convenience sampling method to recruit medical students. We used the WeChat group network to find gatekeepers from each medical university to send the WeChat survey form link to other international students at their universities. A total of 1,219 students submitted their responses. Among them, 12 students did not complete the questionnaire. Therefore, this study used 1,207 responses for analysis purposes. The study covers 15% of the total medical students in China. Due to strict regulations during COVID-19, direct physical access to universities was not possible, thus we relied on online questionnaire methods, which have several advantages, including the fact that these methods do not require interviewers to be present, they are flexible and are suitable for busy people, who are often the educated and well-off (Szolnoki and Hoffmann, 2013).

### Demographic and socioeconomic information

The questionnaire consisted of three parts. First, background information was collected, including age, gender, year of study, marital status, religion, living arrangements, Chinese language proficiency for communication with native people, and teacher cooperation. The nature of the variables was mixed nominal and ordinal. The second part entails the challenges faced by non-native international students and the last section contains depression-related questions.

### Challenges

The challenges considered for this study comprised issued faced such as developing a taste for Chinese food, homesickness, health issues, financial difficulty, living conditions, and ease of transportation, as discussed (Wu et al., 2015; Almurideef, 2016; Jamilah et al., 2020). These questions were dichotomous in nature, and respondents were given two choices: “Yes” or “No.”

### Depressive measures

Depressive symptoms were measured using the CES-D-10, a 10-item Likert scale questionnaire designed by the Center for Epidemiological Studies Depression (Andresen et al., 1994; Björgvinsson et al., 2013; Baron et al., 2017; Trotter T. L. et al., 2019; Trotter R. T. et al., 2019; James et al., 2020; Jamilah et al., 2020). Out of the 10 items, five were about depressed affect, two were about positive affect, and the remaining three were about somatic symptoms. Each question ranged from 0 to 3, where 0 means rarely, while 3 means most of the time. The total scores ranged from 0 to 30, and depressive symptoms cutoff scores were 10 or higher. Cronbach’s alpha for the CES-D-10 was in the acceptable range of 0.812. Descriptive analysis showed the prevalence of depressive symptoms based on background characteristics. Multivariate regression was employed to investigate the influence of the challenges faced by non-native international medical students.

### Data analysis

Statistical package for Social Science (SPSS version 12.0.1) was used to analyze the data. We measured the association between the categorical variables through an association test, the Chi-squared test. The study used binary logistic regression analysis to determine the impact of students’ demographics and living conditions on depression (Outcome binary Variable) (Denham, 2019; Wulandari et al., 2022). Descriptive analysis was employed to learn the demographic information of the respondents. For hypothesis testing purpose, the study employed varying levels of significance (1, 5, and 10%; Browning et al., 2021).

## Results

**Figure 1:**
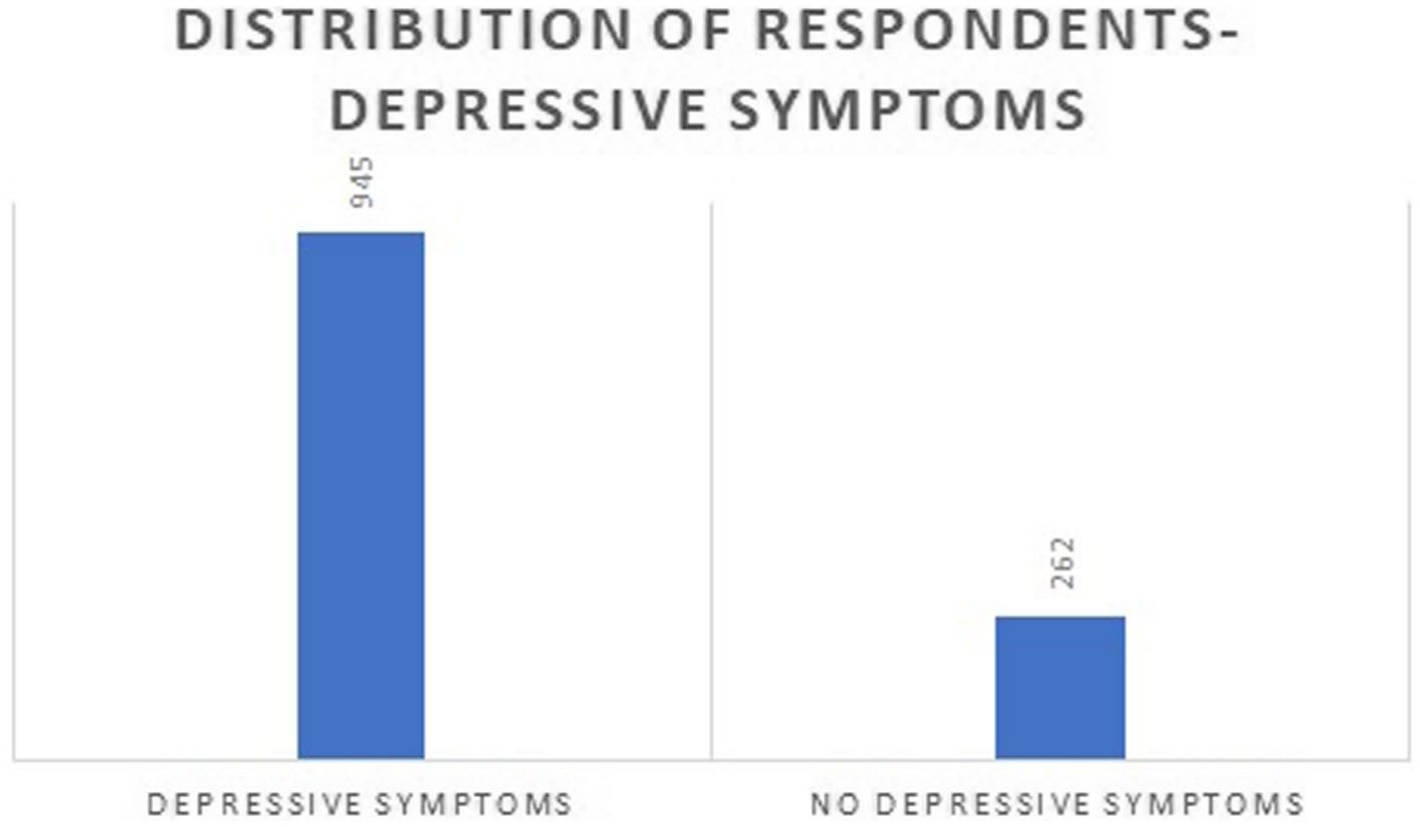
Distribution of Respondents with respect to their depressive symptoms.

Most non-native international medical students were young adults who joined the medical university after college, and 64.95% of the students were between the ages of 18 and 25, as shown in Table 1. The proportion of male students was 52.69, and 44.96% reported experiencing depressive symptoms. In comparison, most female students (76.35%) reported depressive symptoms during COVID-19. The *p*-value shows that gender is strongly associated with depressive symptoms. Figure 1 explains the distribution of respondents depressive symptoms.

**Table 1:** Association between participants background characteristics and depressive symptoms.

Variables	Total *n* (%)	Depressive symptoms	No depressive symptoms	*Q Stat*	*Chi-Square* (d.f)
Total	1,207	945 (78.35)	262 (21.65)		
Age group					
18–25 years	784 (64.95)	651 (83.03)	132 (16.83)	0.71	25.02***(2)
Over 25	423 (35.05)	190 (44.91)	223 (55.08)		
Gender					
Female	571 (47.31)	436 (76.35)	135 (23.64)	0.56	18.32***(2)
Male	636 (52.69)	286 (44.96)	350 (55.03)		
Religious					
Yes	943 (78.12)	113 (11.98)	830 (88.01)	−0.71	14.32**(2)
No	264 (21.88)	118 (44.69)	146 (55.30)		
Marital Status					
Married	144 (11.93%)	90 (62.5)	54 (37.5)	0.76	19,63***(2)
Single	1,063 (88.07)	191 (17.96)	872 (82.03)		
Year of Study					
1st year	663 (54.92)	578 (87.17)	85 (12.82)	−0.89	16.32**(8)
2nd year	301 (24.93)	225 (74.75)	76 (25.24)		
3rd year	144 (11.93)	36 (25.00)	108 (75.00)		
4th year	52 (4.30)	41 (48.8)	43 (51.2)		
Intern	47 (3.89)	14 (46.7)	16 (53.3)		
Living arrangement in China					
Independent living	446 (36.95)	4 (25.0)	12 (75.0)	−0.75	12.01*(4)
Living with friends	580 (48.05)	80 (47.1)	90 (52.9)		
Living with unknown	181 (14.99)	11 (78.6)	3 (21.4)		
Communication in Chinese language					
Not good	685 (56.75)	569 (83.07)	116 (16.93)	−0.89	25.01***(8)
Basic	132 (10.93)	95 (71.96)	37 (28.03)		
Fair	125 (10.35)	81 (64.8)	44 (35.2)		
Good	167 (13.83)	41 (24.55)	126 (75.44)		
Excellent	98 (8.11)	12 (12.24)	86 (87.75)		
Study-related problems					
No	904 (74.89)	154 (17.03)	750 (82.96)	−0.89	14.32**(2)
Yes	303 (25.10)	239 (78.87)	64 (21.12)		
Teacher and student’s academic interaction					
Fair	941 (77.96)	66 (7.01)	875 (92.98)	−0.55	23.81***(4)
Full	188 (15.57)	10 (5.31)	178 (94.68)		
Poor	78 (6.46)	64 (82.05)	14 (17.94)		

*** 1% level of significance.

** 5% level of significance.

* 10% level of significance.

The results showed the association of the aforementioned factors with depression. The results revealed that age has positive associations with depression (0.71) as shown in table one. The overall percentage of single students is high when compared to married students and the Chi-Square test value of *p* (0.00) showed that marital status significantly matters in influencing depression. Furthermore, it was found that married students were more likely to be depressed during COVID-19. In terms of religion, the Q-stat (−0.71) showed that students adhering to a religion were less likely to be prone to depression when compared to those who did not have any religion. The Chinese language is among the most difficult languages of the world and newcomers into the country are more likely to struggle linguistically. The results also found that 1st year students are more likely to face depression compared to their seniors studying in the 2nd year of the programs. Notably, students who have spent more time in China are more familiar with the culture and will be aware of online stores that they can buy necessary things from. Therefore, we argue that senior students have lesser rates of depression as compared to junior or first year students.

Living conditions also contributed to the depressive symptoms of non-native international medical students, as it was found that students living independently or with friends were less depressed than those living with strangers. As in many universities in China, international non-native international students share apartments with native students. It is possible that due to language deficiencies they may be unable to convey their problems and issues to others, resulting in depressive symptoms.

Student and teacher online interactions during this critical time showed that where teachers had poor interactions with students, students experienced higher levels of depressive symptoms, although this percentage was relatively low (less than 10% of the whole sample).

The findings of the multivariable binary logistic regression analysis are presented in Table 2. Results revealed that international students with challenges communicating in the Chinese language were 2.67 (95% confidence interval (1.54–3.87)) times more likely to be depressed than those with good language skills. Similarly, students who did not develop a taste for Chinese food were 1.07 times more depressed than those who developed a taste for Chinese food. Students with accommodation problems during COVID-19 were 1.23 times more likely to face depressive symptoms but insignificantly. Because the situation of COVID was initially worse in China, as the outbreak had started in China, international students with homesickness faced 2.55 times more depressive symptoms during COVID-19, which remained a significant factor for depressive symptoms among international medical students. As is evident, international students who suffered health issues during the pandemic were 3.58 times more depressed (95% CI: 1.67–5.85) during COVID-19 compared to healthy international students. Financial worries were a cause for concern for everyone during the pandemic, and international students were no exception. The results revealed that students who faced financial issues during the pandemic experienced 2.18 times the rate of depressive symptoms as those who had no financial issues. Limited mobility also contributed to international students’ depressive symptoms, as students with mobility issues suffered twice the depressive symptoms as those who could move around and had access to transportation.

**Table 2:** Challenges and depressive symptoms (multivariate binary logistic regression results).

Challenges faced	*p*-Value	OR 95% CI
Communicate in Chinese language		
No		__
Yes	0.00***	2.67 (1.54–3.87)
Problems adapting to local Chinese food		
No		__
Yes	0.0653*	1.07 (0.67–2.54)
Accommodation problems		
No		__
Yes	0.69	1.23 (0.30–3.63)
Homesickness		
No		__
Yes	0.001***	2.55 (1.12–4.04)
Health problems		
No		__
Yes	0.00***	3.58 (1.67–5.85)
Financial difficulty		
No		__
Yes	0.045**	2.18 (1.37–3.10)
Mobility issues		
No		__
Yes	0.451	0.11 (0.45–2.92)
Model summary		
pseudo-R2 (Nagelkerke’s R2)	14.9%	
Cox & Snell and Nagelkerke	11.4%	

*** 1% level of significance.

** 5% level of significance.

* 10% level of significance.

## Discussion

The study reveals the influence of challenges faced by non-native international medical students on rates of depression during COVID-19. The study used the CES-D scale to measure depression symptoms and found that 78.35% of the students suffered from depression. The results revealed that students studying in the 1st year face more challenges, as they are new to the country and are not as familiar with the language, culture, and places, such as hospitals, restaurants or supermarkets, compared to students in their 2nd or 3rd year. Similarly, it is found that depression symptoms are higher in females than males and the results are in agreement with those of Naz et al. (2017) and Ibrayeva et al. (2018). One strand of literature reported that females reported higher levels of stress than males when there was no tangible crisis (McLean and Anderson, 2009). Interestingly, Andresen et al. (1994) reported that females experienced more stress than males during an earthquake. This shows that a crisis such as COVID-19 may deepen this discrepancy as suggested by Debowska et al. (2020). Our findings also support the results of these studies.

COVID-19 disrupts the finances of individuals, and students are no exception. Financial issues are considered an important source of stress among students (Aselton, 2012). They are also a significant factor for depression among international medical students during COVID-19. Garbee et al. (1980) and Eskanadrieh et al. (2012) had similar findings.

Student-faculty interactions are always fruitful and result in students’ better performance (Springer, 1994). It is found that faculty-student interactions that provide support and motivation are important to control levels of depression among students.

Although many students equipped themselves with basic Chinese language skills before coming to China, it may be insufficient for day-to-day communication. Even in clinical work, they may not be able to effectively communicate with patients, which can result in depressive symptoms. As China took strict actions against COVID-19 and locked the whole country down, non-native international students experienced more stress. As they could not communicate with the local people, they were not allowed to go outside. Accordingly, the language barrier became the most prominent and significant source of depressive symptoms among international students.

As China is a desired destination for many international students, the Chinese government should take several steps to accommodate international students, accelerating globalization in China. The Chinese government has already taken several steps to maintain the confidence of existing students in China by providing them with vaccinations on a priority basis and free of charge (Zhou et al., 2021). The Chinese government must manage existing international students’ depressive symptoms due to their limited mobility during COVID-19, otherwise, it would be an added challenge along with COVID-19.

However, this study has a few limitations. First, it was a cross-sectional study that covered only international medical students in China. Future studies may include faculty members to measure and improve students’ performance. Second, this study did not include the nationalities of the respondents; further studies might include nationalities and report regional findings. Furthermore, forthcoming studies may include comparing native and non-native international medical students’ depression levels during COVID-19 in China. Future studies may consider students in other disciplines to explore the differences in mental health issues in detail. As this study only explored the general academic issues, future studies may consider the clinic work experience to elaborate on it more.

Despite these limitations, this study is among the few studies that have addressed language barriers as a source of depressive symptoms during the particular circumstances of the COVID-19 pandemic. Chinese diplomatic missions worldwide can provide short language courses and communicate cultural information while granting study permits. The hosting institutions may offer a platform where new students can interact with alums and already enrolled students so they can discuss potential challenges and prepare themselves before entering China. In this way, students would be well-equipped and more aware of potential challenges before moving into the country.

## Conclusion

This study examines the struggles faced by non-native international medical students studying in China during the COVID-19 pandemic. The findings showed that several factors influence students’ mental well-being while studying medicine during COVID-19. Multiple factors contribute to the students’ depressive symptoms, including language barriers, accommodation struggles, and food adaptability issues. The Chinese government’s strict actions against the pandemic protected international students from health issues, but the language barrier contributed significantly to students’ depressive symptoms during the pandemic. The corresponding diplomatic representatives must assist their students by offering short language courses to their student community before they move to foreign countries. These language skills would help them to survive complex situations faced by international students during COVID-19.

## Data availability statement

The raw data supporting the conclusions of this article will be made available by the authors, without undue reservation.

## Ethics statement

The studies involving human participants were reviewed and approved by an Ethical Committee at the Fujian University of Technology (Ref. No FU: 0020–915). The patients/participants provided their written informed consent to participate in this study.

## Author contributions

The idea was initiated by GX, who worked on the conceptualization of the idea and assisted in the collection of data in his neighboring cities. Initially, the draft was written by MA. The questionnaire was designed and collected by WZ. RR worked on the methodology section of the document and proofread it. The analysis of the data was performed by MN, who was also responsible for managing the software used for the analysis. All authors contributed to the article and approved the submitted version.

## Funding

The research was funded by the 13th 5-year plan in 2018 of General Project of Philosophy and Social Sciences of Guangdong Province (Project No: GD18CSH02) and General Project of Guangzhou Philosophy and Social Science Development (Project No: 2018GZYB134).

## Conflict of interest

The authors declare that the research was conducted in the absence of any commercial or financial relationships that could be construed as a potential conflict of interest.

## Publisher’s note

All claims expressed in this article are solely those of the authors and do not necessarily represent those of their affiliated organizations, or those of the publisher, the editors and the reviewers. Any product that may be evaluated in this article, or claim that may be made by its manufacturer, is not guaranteed or endorsed by the publisher.

## Footnotes

^1^ https://en.moe.gov.cn/cooperation_exchanges/201506/t20150626_191373.html
